# Re-designing materials for biomedical applications: from biomimicry to nature-inspired chemical engineering

**DOI:** 10.1098/rsta.2018.0268

**Published:** 2018-12-24

**Authors:** Ayomi S. Perera, Marc-Olivier Coppens

**Affiliations:** 1 Centre for Nature Inspired Engineering, Department of Chemical Engineering, University College London, Torrington Place, London WC1E 7JE, UK; 2 Department of Chemical and Pharmaceutical Sciences, Kingston University London, Penrhyn Road, Kingston upon Thames KT1 2EE, UK

**Keywords:** nature-inspired engineering, biomedicine, biomedical materials, biomimetics, bioinspired

## Abstract

Gathering inspiration from nature for the design of new materials, products and processes is a topic gaining rapid interest among scientists and engineers. In this review, we introduce the concept of nature-inspired chemical engineering (NICE). We critically examine how this approach offers advantages over straightforward biomimicry and distinguishes itself from bio-integrated design, as a systematic methodology to present innovative solutions to challenging problems. The scope of application of the nature-inspired approach is demonstrated via examples from the field of biomedicine, where much of the inspiration is still more narrowly focused on imitation or bio-integration. We conclude with an outlook on prospective future applications, offered by the more systematic and mechanistically based NICE approach, complemented by rapid progress in manufacturing, computation and robotics.

This article is part of the theme issue ‘Bioinspired materials and surfaces for green science and technology’.

## 1. Introduction

### (a) Nature-inspired chemical engineering (NICE)

Nature is replete with examples of processes that, through evolution, have been perfected over the ages, to effectively use energy, matter and space, and to sustain life. These processes have proven resilient to time-tested environmental changes, and thus are able to recover and rebuild when disturbed or damaged. An extraordinary feature of natural processes is their ability to transcend barriers of scalability, while maintaining efficiency [1]. Together with other desirable traits, such as robustness, dynamic self-organization and self-healing ability, these systems embody ideal characteristics that man-made formations strive for, yet often fail to achieve.

**Figure 1.**
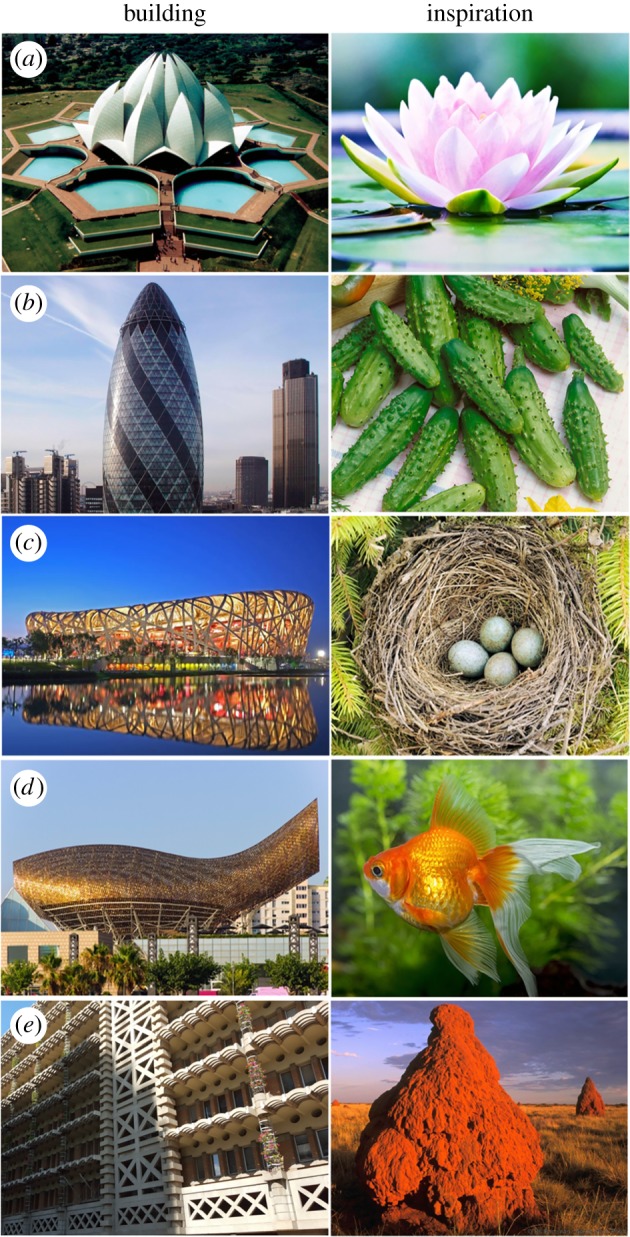
Buildings inspired by nature. (*a*) The lotus temple in India, inspired by the lotus flower; (*b*) the Gherkin in London, UK, inspired by gherkins; (*c*) the Beijing national stadium in China, inspired by birds' nests; (*d*) the Olympic pavilion in Barcelona, Spain, inspired by gold fish; (*e*) and the Eastgate development in Zimbabwe, inspired by termite structures. (Online version in colour.)

The concept of drawing inspiration from nature for the design and engineering of new materials and structures is not new. Since ancient times, man has been inspired by structural wonders found in nature. Architecture is the most prominent field that benefitted from this notion. The remarkable constructions by Eiffel [2], Gaudí [3] and Paxton [4] are examples where natural structures have provided inspiration for unique and enduring man-made creations. Other examples around the world include the lotus temple in India, The Gherkin in London, the Beijing national stadium in China, the Olympic pavilion in Barcelona and the Eastgate development in Zimbabwe, to name a few [5,6] (figure 1). Some of these examples, like the lotus temple, however, mainly mimic their biological counterparts in appearance, with less attention to nature-inspired function.

**Figure 2.**
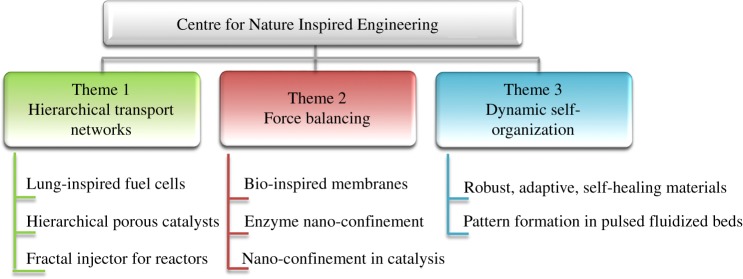
The broad scope of application of the NICE approach, as demonstrated by some of the work conducted at the UCL Centre for Nature Inspired Engineering (CNIE) [1]. (Online version in colour.)

In contrast, nature-inspired design goes beyond structural or aesthetic similarities, and delves into the mechanistic and physico-chemical features of natural systems. The Centre for Nature Inspired Engineering (CNIE) at UCL draws inspiration from such natural processes to create innovative solutions to engineering challenges. In particular, we attempt to address some of our grand challenges in energy, water, functional materials, health, and living space via nature-inspired design [1]. Our research is currently based around three themes, corresponding to three fundamental mechanisms, of wide applicability: (i) hierarchical transport networks, (ii) force balancing and (iii) dynamic self-organization (figure 2).

The goals of this paper are to discuss key issues related to nature-inspired material and process design: (i) Provide clarity on defining nature-inspired design, versus imitation (bio-mimicking) and bio-integrated design, (ii) Offer perspective on the advantages of the NICE approach in (re-) designing new and advanced materials, and (iii) Discuss the broad scope of application of this approach, using examples from literature, with particular focus on biomedical applications.

### (b) Inspiration, imitation and integration: three ways to connect nature to design

In recent years, there has been increased interest within the scientific community in redesigning materials and processes, based on biological or other natural analogies. These studies belong to three distinct categories, based on how the natural component is used: (i) nature-inspired, (ii) nature-mimicking or (iii) nature-integrated design. As most of such studies centre around biological organisms, these definitions also appear in the literature as ‘bio-inspired’, ‘bio-mimicking’ or ‘bio-integrated’ design. The term ‘nature’ infers a broader definition, where features of non-living natural systems are also included, in addition to living systems, for materials and process design. It is important to understand the distinctions between these terms, in order to effectively use each methodology, as the terms are often misrepresented or misinterpreted. The following paragraphs attempt to define each distinct category, clarify what nature-inspired engineering really entails, and why, we believe, it is more systematically applicable in solving engineering challenges. The term ‘bio-inspired’ has been broadly defined as the use of analogous biological systems to develop solutions for engineering problems [7]. This can be expanded to include non-biological natural systems as nature-inspired design [1].

By our definition, nature-inspired engineering is not based on mimicking nature out of context, or succumbing to superficial analogies, but, rather, on taking a scientific approach to uncover fundamental mechanisms underlying desirable traits. These mechanisms are subsequently applied to design and synthesize artificial systems that borrow the traits of the natural model [8]. Thus, nature-inspired designs may not even superficially or morphologically resemble their natural counterparts, but rather function or behave as such. Such designs adopt some of the features of the systematic model, after suitable adaptation to fulfil the different contexts of nature and technology.

**Figure 3.**
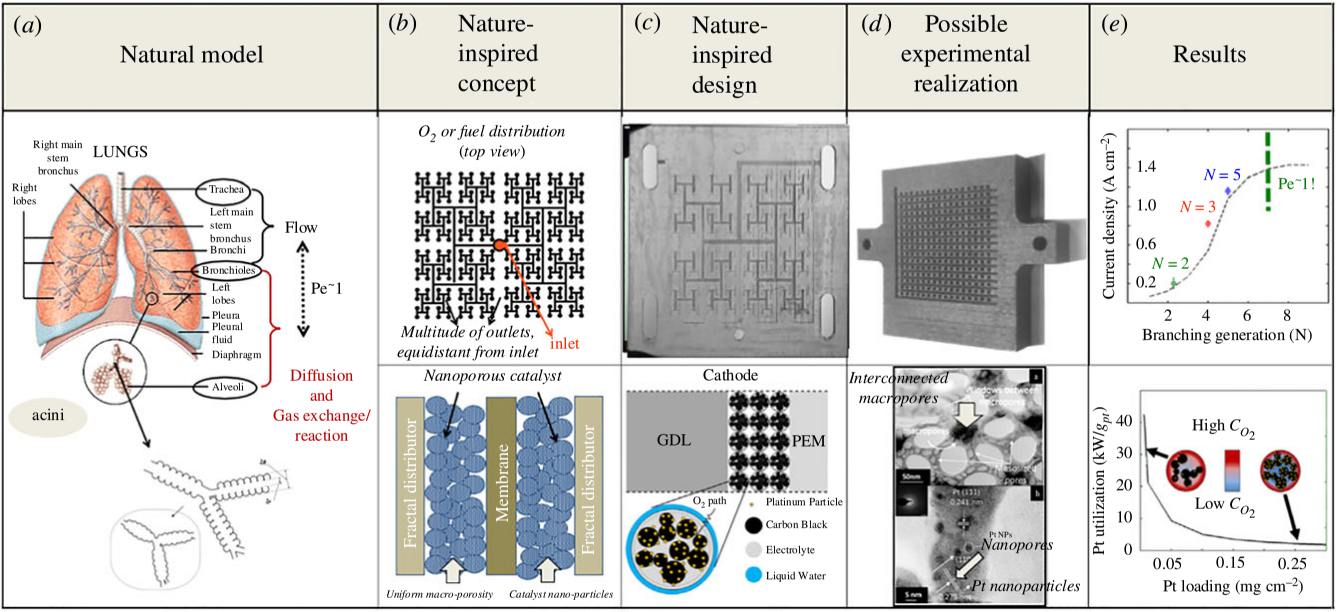
Lung-inspired fuel cell. Illustration of the NICE methodology, which uses fundamental mechanisms underpinning the scalability and efficiency of transport in the lung (*a*) to inspire a concept for transport in a fuel cell (*b*), which is fractal at the macro- to mesoscales of the flow plates (top) and uniform at the meso- to microscale of the porous catalyst (bottom) that is the basis for a new design (*c*), which can be realized thanks to progress in additive manufacturing and chemical synthesis (*d*), which leads to exceptional improvement in fuel cell performance (*e*). This versatile methodology could be applied to trigger innovation in biomedical applications as well. (Online version in colour.)

Figure 2 lists examples of problems tackled by the NICE approach, within the UCL Centre for Nature Inspired Engineering, organized by Theme, following a fundamental mechanism. For example, as illustrated in figure 3, a polymer electrolyte membrane (PEM) fuel cell was redesigned via inspiration from the human lung with its unique hierarchical transport network, with a scalable architecture and dimensions optimized for minimal metabolic energy consumption for breathing [9]. In the lung-inspired PEM fuel cell, the flow channel network for gas transport in the bipolar (current carrying) plates is derived from the fractal geometry of the upper airway tree of the lung, while the porosity distribution of the electrocatalyst draws inspiration from the alveolar sacs, as convection-driven flow gives way to diffusion as the dominant transport mechanism, from bronchi to alveoli, leading to minimal resistance. Dimensions are based on fundamental thermodynamics and physico-chemical principles, rather than straightforward imitation of the lung's appearance. The design is realized using advances in additive manufacturing and materials synthesis. This systematic methodology, from natural model, to nature-inspired concept, design, and realization, is illustrated in figure 3. Compared to traditional designs, this lung-inspired design has great potential for scale up, is more robust and overcomes reactant homogeneity issues in PEM fuel cells. The method has shown to improve energy efficiency and save on expensive catalyst material [10].

Similarly, the field of heterogeneous catalysis has also benefitted from nature-inspired design. By using hierarchical transport networks such as those found in trees as inspiration, new types of catalysts can be developed to overcome diffusion limitations and optimize transport, leading to more efficient catalyst design [1,11,12]. Tree-inspired fractal injectors facilitate scale-up, and uniformise the flow distribution in multiphase reactors [1,11,12]. The behaviour and properties of wind-swept sand dunes have also been used as inspiration, to induce pattern formation via a pulsed gas flow in fluidized beds, hereby structuring their dynamics, and avoiding maldistribution [13–15]. It is important to note that neither the fuel cells, catalysts nor reactors physically resemble their natural sources of inspiration, but rather express properties or functions of them, and are hence, truly, nature-inspired inventions.

Nature-imitation or mimicking, on the other hand, refers to more straightforward copying of physical and morphological features of organisms or natural systems in product or process design, however it does not take, or insufficiently takes into account, mechanistic features. Imitating nature tends to target a single feature of its natural counterpart, by mimicking its characteristics. Biomimetic robot design is a prominent example, where movement of dogs, birds and insects have been used as inspiration, to guide robot locomotion [16,17]. The behaviours thus adapted include flapping-wing flight, jumping, crawling, wall climbing, and swimming. Great progress has also been made in the development of artificial muscles, propellers and actuators [17]. Other examples include the mimicking of pinecone structure for clothing design to help regulate body temperature [18], the basilisk lizard to design micro-robots that can walk on water [19], and the lotus leaf structure for design of superhydrophobic, self-cleaning coatings [20,21], to name a few.

Nature-integrated or bio-incorporated design is yet another category, which includes living organisms as part of a product or process. The best known cases are those where living components are integrated into buildings to fulfil key functional purposes. Germany's ‘algae house’ or BIQ building in Hamburg is a well-known example, where living microalgae are incorporated into the walls [22]. The microalgae are grown in transparent surfaces and harness sunlight to provide renewable energy to the building. The Netherlands' Sportplaza Mercator is another example, which incorporates lush vegetation to create living walls on its breath-taking façade [5].

### (c) Why should we look to nature for answers?

Scientific research becomes increasingly challenging, as we attempt to tackle more and more complex technical problems, and answer deeper scientific questions, regardless of the advances made in synthesis and manufacturing. Often times, there are too many variables to consider for a comprehensive analysis of the problem at hand, with limited time and resources. Developing methodologies to optimize the management of time, materials, energy cost, man-power, instrument availability and maintenance cost is crucial in research. Empiricism goes only so far. Brute-force computational approaches have their limitations, and do not necessarily provide understanding. If there were to be a blueprint or guideline of strategies and methodologies that could increase the efficiency of research and development, and lead to more sustainable solutions, its impact on scientific advancement would be immeasurable. That is where nature comes in. Natural systems are full of characteristics and mechanisms that can be studied and used to give us clues to the solution of critical problems [8,23,24].

**Figure 4.**
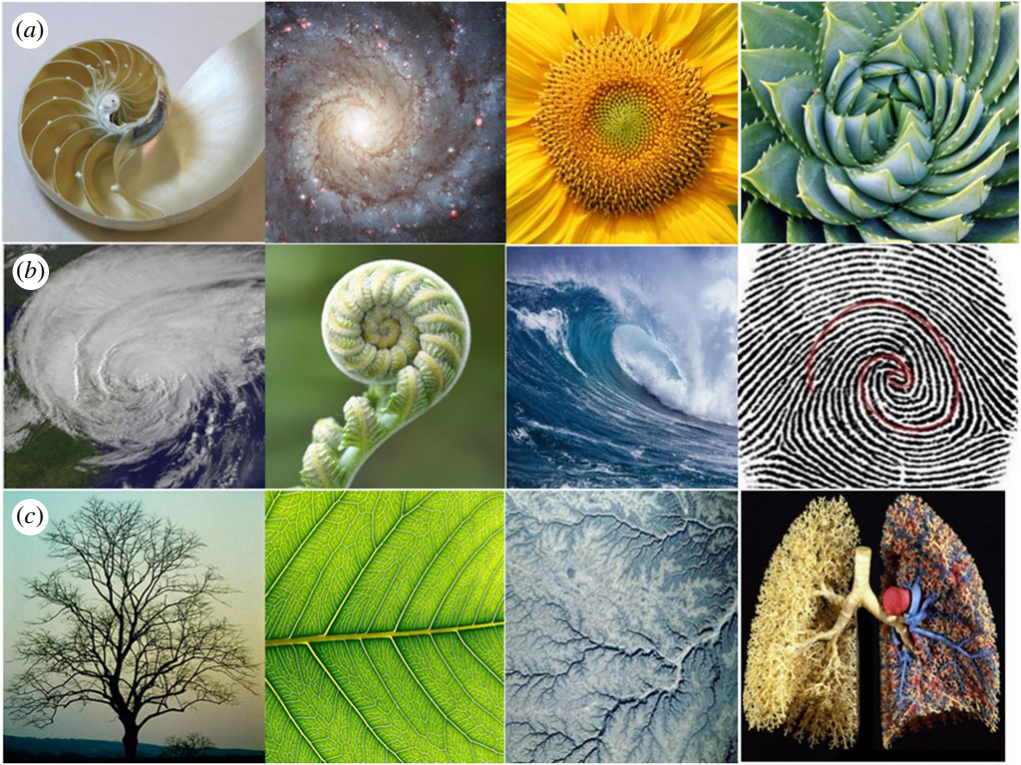
Examples of patterns in nature, including the golden spiral, the golden ratio and fractal self-similar structures. From left to right, (*a*) a nautilus shell, a galaxy, a sunflower, a desert plant; (*b*) a storm formation, a fern bud, an ocean wave, a finger print; (*c*) fractal properties in a branched tree, a leaf, river bed formation, a cast of human lungs. (Online version in colour.)

Careful examination of natural systems reveals certain patterns that are key to underlying structures, which, in turn, may relate to desirable properties. One example, arising from dynamic self-assembly, consists of regularly striped patterns on dunes, altocumulus clouds and Rayleigh–Bénard convection rolls. Another is related to the Fibonacci sequence and the golden spiral [25]. Fibonacci, a twelfth century Italian mathematician, described a sequence of numbers, now known as the Fibonacci sequence. The sequence starts with 1, and each next number is the sum of the two previous numbers, and, hence, goes as: 1, 1, 2, 3, 5, 8, 13, 21, 34, 55, 89, 144, 233, etc. [26,27]. The ratio between subsequent numbers in this series approaches what is known as the golden ratio. This ratio is believed by many to represent unique functional and aesthetic ideals in nature. It corresponds to the ratio between the lengths of the larger and smaller sides of a rectangle, and is approximately equal to 1.618. Such a rectangle is called a golden or a perfect rectangle. If a perfect rectangle is divided into squares based on the Fibonacci numbers, and each square is crossed with a tangential arc to connect them, a spiral takes shape, which is called the golden spiral. It has been noted that the Fibonacci numbers, the golden ratio and the golden spiral are universally ubiquitous in nature (figure 4) [25,28]. The nautilus shell, a galaxy, seeds of a sunflower, cactus leaves, a storm formation, a fern bud, a sea wave and a fingerprint all contain examples of the golden spiral in nature. Moreover, the branching of trees, leaves, riverbeds and even the human lung contain fractal divisions that relate to the golden ratio. In many cases, such features result from optimization processes of energy, matter and space in natural systems, sometimes by self-organization, sometimes by evolution. Observing such patterns in nature helps us to describe them mathematically, and start looking for underlying reasons (are they satisfying some optimality criterion under constraints?) as a basis for developing a systematic approach to understanding natural materials, and processes. Such deeper insight can lead to meaningful and scientific, nature-inspired engineering solutions.

Cataloguing characteristics in the hugely diverse systems that constitute nature is not easy. From a materials science viewpoint, according to Arzt and Meyers, biological systems have six major characteristics that lead to remarkably advantageous properties: (i) self-assembly, (ii) multi-functionality, (iii) hydration, (iv) evolution and environmental constraints, (v) synthesis at ambient conditions, and (vi) hierarchical structures [29,30]. Each of these characteristics is particularly prominent in biological systems, as opposed to most synthetic counterparts today. For example, self-assembly in biological structures broadly refers to them being assembled from the bottom-up, rather than top-down, as, e.g., in lithographic techniques. This ensures continuous growth, as there is no overriding scaffold that terminates top-down growth.

Components of biological systems usually serve more than one purpose, so as to optimize resources, and are, hence, multifunctional. The properties of such structures are drastically influenced by the amount of water they contain, thus, hydration is a key feature. Some of the most widely used elements in man-made materials are iron, aluminium and copper. All of these require high temperatures for processing, leading to high production costs and energy requirements. However, years of evolution, environmental constraints and limited resources have constrained nature to build robust, functional biological structures with only a few key elements, predominantly carbon, oxygen, hydrogen, nitrogen, calcium, phosphorous and silicon. These elements are put together at ambient temperature and atmospheric conditions, in building those structures. Finally, the property of hierarchy is omnipresent in biology, where structures consist of multiple scales, each of which deliberates a distinctive feature, as in bones or tendons. In some cases, the hierarchy contains a fractal scaling range, in which features repeat themselves repeatedly under magnification, e.g. by self-similarity (figure 4*c*).

Taking inspiration from such features can lead to the development of new materials and technologies with minimal resource consumption and environmental impact. As a result, the reach of nature-inspired design in science and engineering is vast, and expanding. Herein, we focus on one key area: that of biomedical applications.

## 2. Biomedical applications

In biomedical design, engineering principles are applied to medicine and biological systems for the purpose of designing applications for healthcare. This includes diagnostics, therapeutics and analysis. Not seeking to be comprehensive, but rather illustrative, the following sections will discuss a few selected biomedical examples of nature-inspired design, including membranes and coatings for tissue engineering scaffolds, as well as implants and support materials. Attention will be given to a few key sources of inspiration, with particular focus on mollusc nacre, and some other selected examples that were chosen because of their remarkable features, as well as broad applications.

### (a) Tissue regeneration, implants and support materials

#### (i) Nacre-inspired materials

As one would expect, the development of new types of materials for tissue engineering has been significantly influenced by biological materials [30]. Bio-materials, such as mammalian bones, crustacean shells and reptile skins have unique properties that are intimately associated to their structure. These structures usually consist of elaborate, hierarchical arrangements and interactions, across multiple length scales, giving them superior mechanical strength, adaptability and self-healing ability [30,31]. Associated properties are often the result of a combination of two distinct components: a ‘hard’ component, consisting of bio-minerals, such as calcium carbonate, hydroxyapatite or silica, and a ‘soft’ component, consisting of organic matter, such as collagen, elastin or cellulose.

**Figure 5.**
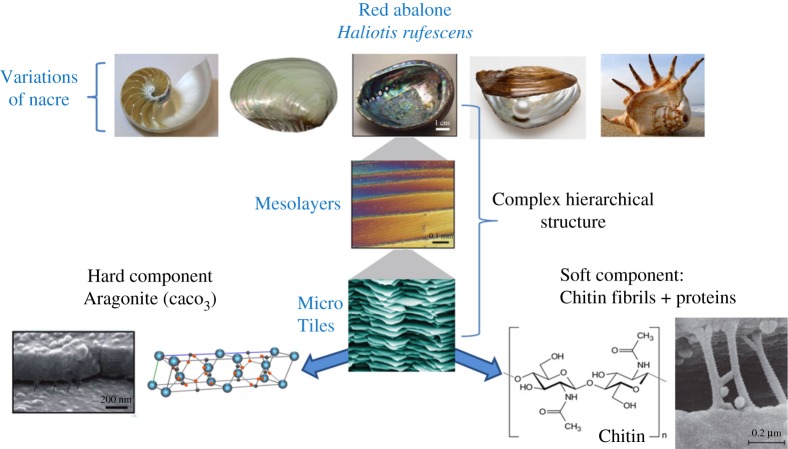
Various mollusc species containing nacre in their inner shell, and the complex and hierarchical structure of nacre, as described via the red abalone shell [30,33,34]. Modified with permission from references [27,35,36]. (Online version in colour.)

**Figure 6.**
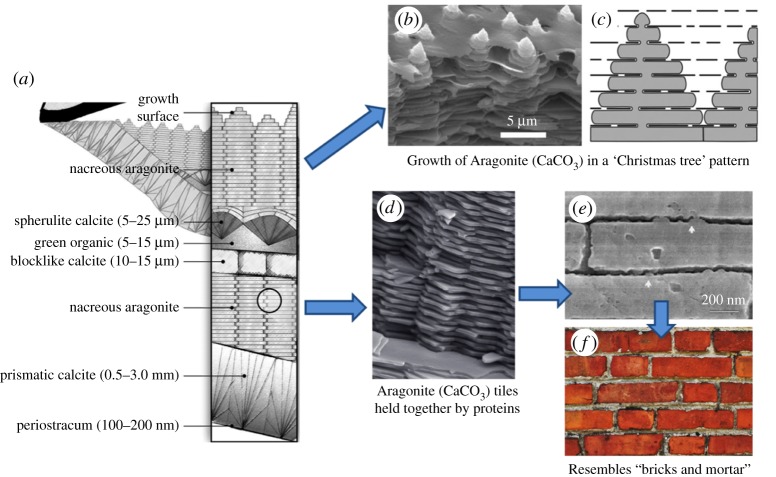
A closer look at the layer-by-layer arrangement of CaCO_3_ plates and organic matrix in the nacre of the red abalone shell. (*a*) Vertical section of the nacre outer edge of the shell and mantle [32], (*b*) SEM image of a growth surface of nacre, showing the ‘Christmas tree’ growth pattern [32,40], (*c*) schematic representation of nacre depicting the growth pattern [32], (*d*) SEM image depicting the layered aragonite plates held together by proteins [36], (*e*) zoomed in SEM image of the cross section of the aragonite plates and proteins [37], (*f*) image of a brick wall. Adapted with permission from references [29,31,37,41]. (Online version in colour.)

Nacre, or the inner shell layer of molluscs, is an example of such a composite, hybrid structure that has been widely used as inspiration in biomedicine [32]. The structure of the red abalone (*Haliotis rufescens*) shell, in particular, has been studied extensively (figure 5) [35]. In general, nacre consists of 95 wt% of aragonite, which is a crystalline form of CaCO_3_ and 5 wt% organic materials, which are proteins and polysaccharides. The aragonite is present as hexagonal plates in a layered arrangement, and the organic matter is present in between the plates, acting as a glue to keep them in place [37]. This arrangement of columnar sheets and tiles is spread across multiple length scales in a hierarchical manner, from nanoscale to macroscale [38]. Moreover, features are also present that enhance mechanical properties, such as mineral bridges [33] and an interlocking mechanism in between plates [36], rotatable nanograins (or nano-asperity) [39], and plastic micro-buckling to decrease strain energy. Due to this ingenious structure, nacre displays superior mechanical properties compared to monolithic aragonite. These include high fracture toughness, high energy absorption, and prevention of crack propagation, while being incredibly light-weight. Nacre also displays continuous growth following a mechanism called the ‘Christmas tree’ pattern, and has the ability to self-heal once damaged, being guided by the organic matrix (figure 6). A horizontal view of the aragonite plates, together with the organic glue, bears a remarkable resemblance to a brick wall, in both structure and function. Thus, nacre is often referred to as ‘nature's bricks and mortar’. These extraordinary features of nacre have attracted attention from material scientists, for the development of novel composite and laminated materials, especially for biomedical applications.

Production of nacre-inspired bioactive materials is a key research area in this field. Nacre-inspired porous scaffolds for bone repair have been established via a combination of strategies, including electrospinning, phase separation, and 3D printing [42]. A composite film consisting of clay nano-platelets and polyimide has been fabricated via a centrifugal deposition process, to form an alternating, layer-by-layer, nacre-like structure [43]. This material displays mechanical properties comparable to bone and has potential applications as bioactive bone replacement or dental graft. A nacre-inspired, layered, freestanding membrane consisting of chitosan, hyaluronic acid and bioactive glass nanoparticles, has been produced as another potential bone replacement material [44]. This material displays enhanced mechanical and bioactivity properties, together with highly tunable physical characteristics, controlled via altering the nanoparticle content.

Nacre's property of combining hard and soft materials in a hierarchical manner has been leveraged to create robust, artificial bone materials, via a sol-gel process [45,46]. In this case, TiO_2_ was polymerized with a flexible polymer (polydimethylsiloxane or polytetramethyloxide), to deposit nano-sized apatite particles on a fine polymer matrix, which was subsequently textured into an intricate 3D framework. These hybrid materials show a Young's modulus and bending strength similar to those of human cancellous bone, together with increased deformability, making them attractive materials for bone grafting. The same inspiration has also been used to create organic-inorganic hybrid ceramic-polymer and alumina-polymer materials with superior mechanical properties [47].

Combination of magnetic nanoparticles (MNPs) with a polymeric matrix is an area that is gaining increased interest for various biomedical applications [41,48,49]. Layer-by-layer assembly of MNPs held together by chitosan and alginate, resembling the structure of nacre, was shown to produce membranes with increased Young's modulus and tensile strength [50]. These materials display magnetic response and adaptive properties that can be triggered by hydration. They were also shown to be more biocompatible compared to non-magnetic counterparts, with increased cell viability, adhesion and proliferation. MNPs have also been used in combination with graphene, to create hierarchical, soft, biocompatible materials, with potential applications as tissue engineering scaffolds and artificial muscles [51].

The development of coatings for biomedical scaffolds and implants has also been inspired by nacre. The main purpose of such coatings is to provide a means to functionalize the surface of biomaterials, in order to induce desired biological responses [52,53]. Multilayered coatings consisting of bioactive glass nanoparticles, chitosan, and catechol functionalized hyaluronic acid, have been developed, inspired by marine mussel nacre [54]. These materials display enhanced mechanical adhesion, and were shown to promote bonelike apatite formation, *in vitro*. In addition, multilayer coatings combining chitosan and bioactive glass nanoparticles, developed via sequential deposition display robustness, together with apatite forming ability, when immersed in simulated body fluid [55]. This methodology has potential in creating scaffolds and implants with complex geometries for orthopaedic applications. Moreover, the approach has been further extended to include antibacterial properties, via incorporation of silver nanoparticles in the coating, which reduces the possibility of infection in implants [56]. Fibres that resemble nacre-like properties have been developed via shear-induced self-assembly of nanoplatelets, and have shown potential in the field of optomechanics, with scalability [57]. It must be noted that there is also a vast literature on synthetic strategies to develop nacre-inspired materials for applications outside biomedicine [46,58–60].

#### (ii) Other bioinspired materials

**Figure 7.**
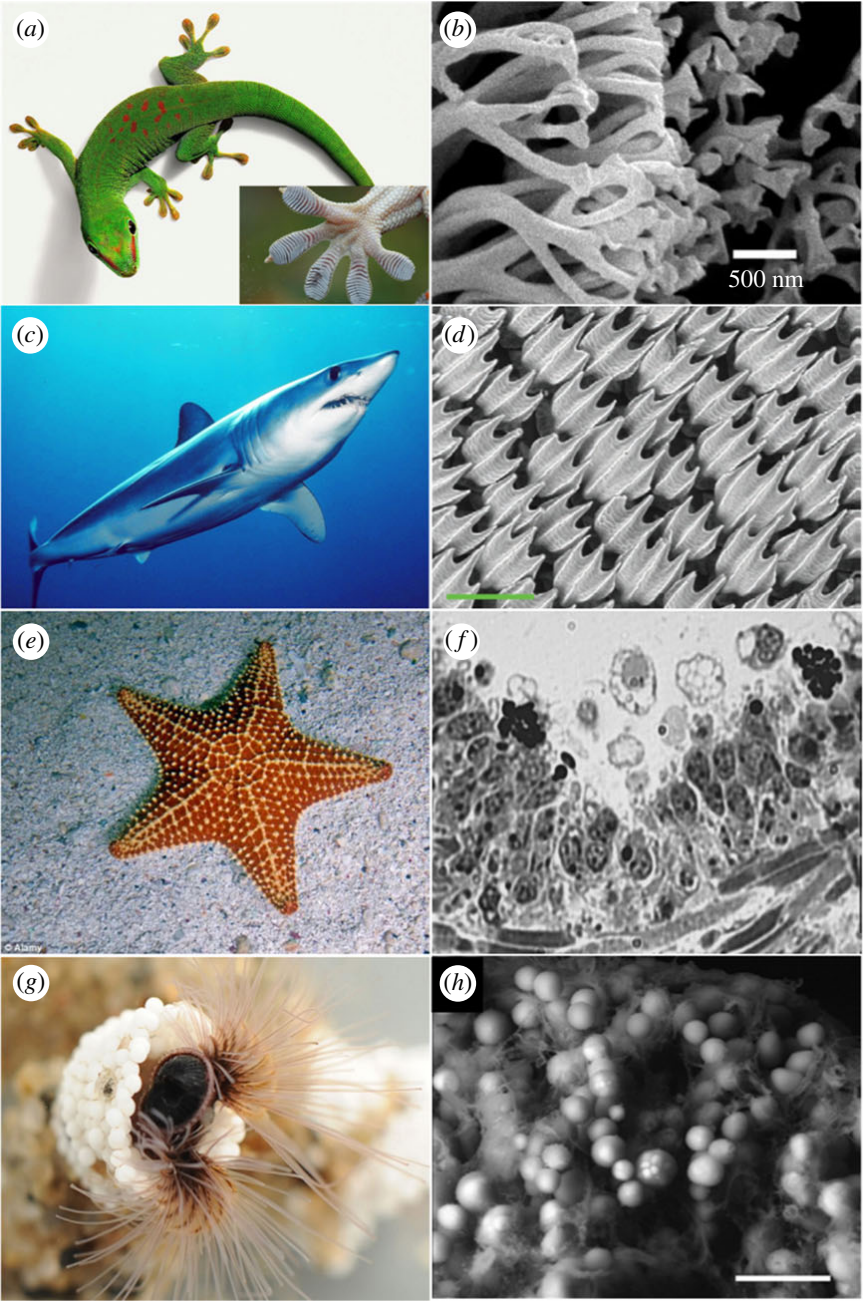
Examples of biological structures that provide inspiration for the design of biomedical materials. (*a*) The gecko's foot, inset—enhanced image of a gecko foot, (*b*) SEM image of the foot hairs of Tokay Gecko (*Gekko gecko*) [61], (*c*) the shortfin mako shark, (*d*) ESEM image of the bonnethead shark (*Sphyrna tiburo*) skin surface [62], (*e*) star fish, belonging to the class of echinoderms, (*f*) optical microscope image of the coelomic epithelium of starfish (*Asterias rubens*) [63], (*g*) the sandcastle worm (*h*) SEM image of the adhesive precursor granules secreted by the sandcastle worm (*Phragmatopoma californica*) [64]. The microscopic images are adapted with permission from references [62–65]. (Online version in colour.)

Many other materials in biology have been used as inspiration to develop innovative, synthetic analogues for biomedicine (figure 7). The gecko foot is a prominent example, studied extensively by material scientists for its remarkable ability to attach strongly to various surfaces and yet be adaptable and recover from mechanical stresses [66,67]. A gecko-inspired, biodegradable and biocompatible adhesive was developed from surface modified poly(glycerol-co-sebacate acrylate), in a nanoscale pillar arrangement, used in combination with a thin tissue-reactive surface [68]. This material has displayed superior adhesion to porcine intestine tissue *in vitro*, and to the rat abdominal muscle tissue, *in vivo*. It is also thought to have potential applications in wound healing and replacement of tissue joints. The development of novel adhesives for non-biomedical applications has also been inspired by the gecko-foot [65,69,70].

The unique structure of shark skin has been used as inspiration to fabricate functional surfaces with advanced properties. An example is a material named as the Sharklet™ micropattern, which has surfaces resilient to bacterial infections, without the addition of any antimicrobial agents [71]. This material has shown potential for use in hospitals and other places where bacteria-borne infections are likely to occur. A synthetic, flexible membrane, inspired by the shortfin mako shark (*Isurus oxyrinchus*), has been developed via 3D modelling and printing [62]. Such discoveries open up possibilities to develop resilient materials for various applications. The remarkable tissue regenerative abilities of Echinoderms have also provided inspiration to achieve advances in the field of tissue engineering [72]. A bio-adhesive material capable of holding bone fractions, and allowing 3D cell alignment, together with the growth of new bone cells, has been developed based on a secretion produced by the sandcastle worm (*Phragmatopoma californica*) [73]. Such adhesives are said to be particularly useful in treatment of craniofacial injuries and trauma. The advanced mechanical properties of insect cuticles have also provided inspiration for the development of a tough, yet lightweight material, which is also biocompatible, biodegradable, and micro-mouldable [74]. This material named ‘shrilk’ has potential to be used as a biodegradable alternative to plastics, replacing a wide range of non-biodegradable products. In addition, the mechanical properties of biomedically important materials such as hydrogels have been dramatically improved, by incorporation of biomimetic nanoparticles [75].

Going beyond the inspiration of animal tissues, technologies that involve customization of living cells [76] or creating artificial cells [77], based on bioinspired methodologies, are emerging as novel promising research areas.

### (b) Drug delivery

The process of drug delivery consists of various engineered technologies for targeted and/or controlled release of therapeutic agents. Traditionally, the main method of delivering drugs to a desired area of the body has included pills taken orally, eye drops, ointments and intravenous solutions. Later on, more sophisticated approaches, using polymeric materials (hydrogels and fibres), vesicles (liposomes and micelles), and chemically modified drugs have been introduced to achieve more target-specific delivery [78,79]. In recent years, with the advancement of nanotechnology, major developments have revolutionized this field, with nanoparticle and liposome loaded drugs that can precisely target specific regions or organs in the body [80]. However, challenges still remain, as most of these new technologies fail to reach clinical expectations. Novel approaches inspired by nature could provide alternative solutions, and lead to precise and sustainable practices in drug delivery. A few relevant examples are discussed below.

#### (i) Nature-inspired methodologies

Polydopamine (PDA) coatings are a key example of a nature-inspired compound, used in various drug delivery applications [81]. These are synthetic pigments derived from a naturally occurring pigment, melanin, and used in multiple biomedical applications. PDA-coated nanoparticles [82,83] and emulsions [84] have demonstrated potential to be used as alternatives to the polymer capsule production method. They have the advantages of being much easier to fabricate, while being highly customizable in terms of physical–chemical properties, to suit specific target sites [84,85]. A mussel-inspired PDA capsule has also been developed by a PDA coating method [86]. These capsules have been used successfully in the intracellular delivery of the anti-cancer drug, doxorubicin. This has been achieved by conjugating the drug with thiolated poly(methacrylic acid) polymer and immobilizing it within the PDA capsule via carrying out thiol–catechol reactions between polymer functional groups and the capsule walls. The system is capable of sustained pH-induced drug release, leading to a stimulus-responsive, nature-inspired methodology for drug delivery.

Recently, non-spherical polymersomes, inspired by the structure of cells, have been synthesized using amphiphilic block copolymers, targeting drug delivery applications [87,88]. The polymers are capable of self-assembling into ellipsoidal shapes, resembling cells, and can be directed to release toxins to cancer cells. The non-spherical shape is said to be far superior in such applications, and previously has been a challenge to achieve, synthetically. With high stability, responsiveness to chemical–physical stimuli, and ability to be functionalized with bio-receptors, these materials are expected to greatly improve the precision and efficiency of cancer targeted drug delivery.

With the advancement of nanoparticles as effective drug carriers, various strategies to improve their performance have also evolved. One critical method is to develop coatings or shields that can improve their biocompatibility. Bioinspired coating strategies are emerging as a dynamic, ‘active’ way of shielding nanoparticles effectively, *in vivo*, rather than the conventional ‘passive’ methods [89]. This is achieved by employing carbohydrates, lipids and proteins, which are often found as natural shields on cells. Hyaluronic acid, which is a polysaccharide, has been coated onto liposomes and used as an intravenous drug carrier [90]. This system has displayed less accelerated blood clearance and side effects in mice compared to conventional, polyethylene glycol (PEG) coated liposomes. Nanoparticles consisting of polysialic acid and modified with hydrophobic groups, as effective carriers of doxorubicin [91]. These particles have displayed low toxicity on non-cancerous cells, yet as much toxicity on cancer cells as free doxorubicin. Sialic acid is a monosaccharide that is found in the body as an amino acid modifier, and the superior performance of the nanoparticle-drug system is attributed to its biocompatibility. The membranes from red blood cells (RBCs) have also been investigated as an alternative to PEG coatings, with various nanoparticles [92–95]. In these studies, the RBC membrane coated particles, through a combination of bio-inspired and bio-integrated design, showed superior biocompatibility properties, such as increased blood circulation and retention times [92,94], decreased macrophage uptake, low toxicity, deceleration in blood clearance [93,95], and higher uptake by the tumour cells [94,95], compared to PEG coated particles. This is owed to the structure of the RBC membranes, resembling those on cells, with lipid bilayer feature and membrane proteins, thus improving the biocompatibility of the delivery system. Drug-loaded nanoparticles coated with peptides derived from the cell membrane glycoprotein CD47, have shown similar traits [96,97].

#### (ii) Biomimetic and bio-integrated approaches

The structures and functions of pathogens, including bacteria and viruses, have been used extensively to develop new types of delivery techniques [98]. A nanogel with virus-like features has been effectively used as a vehicle to deliver doxorubicin to tumour cells, *in vitro* [99]. This system was pH sensitive, and consisted of a hydrophobic polymer core and two layers of hydrophilic shells, in which the outer shell, made of bovine albumin serum, resembled a virus capsid. The inner shell, formed of PEG, acts as glue, linking the core and the outer shell. The core was loaded with doxorubicin, along with a polymer. This nanogel displayed a behaviour similar to that of a virus, where it actively infects tumour cells in a receptor-dependent manner, then destroys the cells, and migrates to neighbouring cells to repeat the process again. Polymer micelle assemblies called ‘filomicelles’, which resemble filamentous viruses, have also been effectively used to deliver the anti-cancer drug paclitaxel into mice tumours [100]. The filomicelles displayed blood circulation times that were ten times longer than their spherical counterparts, and exhibited high anti-cancer activity. This study provided further evidence that the shape of drug delivery vehicles plays a role in their effectiveness, and that shapes mimicking those in natural biological systems are more desirable.

Another approach is to use particles that mimic the shape, structure and functions of cells. Synthetic particles that resemble RBCs are a growing area of interest in this field [101]. RBC-mimicking particles with the ability to carry oxygen and move through capillaries smaller than their own size, have been developed by using hollow polystyrene spheres [102]. These particles are also able to encapsulate drugs and imaging agents, much like RBCs. Hydrogel microparticles resembling RBCs in size, shape and elasticity, have also shown promise as highly stable and biocompatible delivery vehicles [103]. The biodistribution and blood circulation properties of these particles were increased significantly, compared to conventional microparticles, by customizing their elastic modulus in the physiological range. A combination of mimicking both the shape and structure of RBCs has also resulted in enhanced blood circulation times, found by another recent study [104]. In this case, cellulose microparticles were coated with a natural RBC membrane, collected from mouse blood, as a method to enhance biocompatibility. These studies indicate that combining the functions of natural RBCs, together with the versatility of synthetic particles, can lead to the development of a highly effective class of delivery agents. A better understanding of the underlying principles would help to move from biomimetic to truly bioinspired designs, using the systematism of the NICE approach.

In some cases, genetically engineered pathogens have been used as biocompatible alternatives to conventional drug delivery methods. A phase I clinical trial has been successfully conducted using the genetically modified *Lactococcus lactis* bacteria for effective mucosal delivery of immunomodulatory proteins [105]. Patients with a chronic intestinal disease called Crohn's disease were given the above remedy and showed far less systemic side effects, compared to a placebo group. The same bacteria, engineered in a different way, have been used successfully in a mouthwash solution, to treat oral mucositis [106]. Genetically modified *Salmonella typhi* has also been used as a vector to target tumour cells and to effectively deliver a multidrug-resistant gene, MDR1 siRNA [107]. This system has displayed efficient cytotoxicity *in vitro*, as well as suppression of tumour proliferation *in vivo*, when administered orally, in mice. Similarly, *Salmonella typhimurium*, genetically modified with the transcription factor STAT3 SiRNA, has displayed significant anti-cancer activity in mice [108]. In this case, inhibition in tumour growth, in the liver and spleen in mice, along with prolonging the lifespan of prostate tumour induced mice, have been observed, and compared to those treated with the attenuated, unmodified bacterial strain. Although all of the above studies have shown promise, the use of living pathogens *in vivo* comes with inherent safety concerns, which must be carefully considered in designing clinical trials. One objective could thus be to develop bioinspired designs that maintain the biocompatibility of the bio-integrated approaches.

### (c) Other applications: novel composite materials and robotics

Characteristics of biomineralization have been used to create synthetic microenvironments that allow customization of artificial crystallization [109]. This development indicates that certain aspects of crystallization that are generally difficult to influence, such as the precise localization of particles, nucleation density, crystal sizes, crystallographic orientation, morphology, polymorph, stability, and architecture, could be tailored via a nature-inspired approach. Alumina platelets coated with MNPs, inspired by the layer-by-layer composite fabrication approach used by cells, have been magnetically aligned to create highly customizable, robust materials for biomedical and other applications [110]. Due to the advanced material properties, achieved by this approach, such as locally controlled texture and high mineral content, various complex-shaped composites can be created, which are otherwise impossible to engineer using conventional technologies. Another example uses 3D printing to create complex structures of bone-inspired materials with high energy absorption capabilities, targeting multiple applications. A hard shell-soft core composite material (similar to bone) was moulded into complex architectures using 3D printing, and shown to have enhanced, tunable mechanical properties to suit specific applications [111]. Such materials have far superior mechanical strength, compared to pure stiff polymeric composites. A robocasting 3D printing technique has also been used to develop nacre-inspired ceramic composite materials with complex structures, paving a new way to obtaining unprecedented scaffold microstructures [112].

Some of the most promising biomedical applications of nature-inspired engineering are in the field of robotics. A recent issue of *Nature Materials* has been devoted to this [104–108]. 3D printed soft robots are of particular interest, which can either be integrated with human tissues or used as biomedical devices [113]. These can mimic the movements of animals, and allow for responsive, adaptive deformation and movement, which are highly beneficial properties for biomedical applications. Another category is origami robots, inspired by the natural folding mechanisms seen in proteins, intestines, etc. [114]. These robots are built according to a top-down approach, with smart material actuators embedded within, and are capable of folding, to achieve a wide range of robot morphologies with complex functionalities. Origami robots embody many characteristics of living organisms, such as self-assembly, movement, shape change and sensing, making them excellent candidates for future medical devices. For example, ingestible origami pills have been developed that can deploy inside the stomach and perform various functions, such as patch wounds, deliver drugs or remove foreign objects. Such technologies pave the way towards non-invasive, incision-free techniques to perform surgery [115]. Locomotion has also been the key focus in the development of magnetic micro-robots, inspired by the movements of microorganisms [116]. The robots consist of soft polymeric materials, together with magnetic micro- and nanoparticles, and use flagella and filament-inspired components to achieve self-propulsion, in response to external stimuli, such as magnetic and electric fields. They can also exhibit collective behaviour over longer periods of time. These have great potential in the advancement of surgical techniques and targeted drug delivery.

Finally, hydrogel ionotronics is another rapidly evolving field, where hydrogels are incorporated with mobile ions and electrons, and can act as hybrid, stretchable, transparent ionic conductors similar to an ionotropic device [117]. These could serve to develop artificial muscles, skins and axons, along with various other applications.

## 3. Outlook and conclusion

It is striking that many of the discussed examples are based on a combination of mechanistic insights (bioinspired) with biomimicry, while the integration of biological components may be chosen for reasons of biocompatibility or to directly use desired features of the biological ingredients. Materials scientists continue to develop novel nature-inspired and biomimetic materials with advanced properties, but the full potential of learning lessons from nature has yet to be realized.

This is where, we believe, the nature-inspired chemical engineering (NICE) approach has a lot to offer. It is systematic, versatile and based on fundamental mechanisms, rather than superficial analogies. The versatility of the approach is partly due to the ability to translate a validated application from one field to another, promoting lateral thinking. The systematism is reflected in the step-by-step process of deriving mechanisms from the model natural system (which underpins the desired properties) to create a nature-inspired concept, which leads to a proposed nature-inspired design, and its implementation, cognizant of the differences between the natural context and that of the engineering application. These unique characteristics of the NICE approach, already successfully applied across many other application areas, suggest that biomedical research could greatly benefit from it. Indeed, most examples discussed here do not follow such a systematic methodology, and there is a gap between materials science, engineering, and the medical context of the application. A product-oriented engineering mindset is required to close this gap.

The NICE approach provides opportunities to redesign materials and processes, in a capacity that supersedes conventional technologies, and leads to sustainable solutions to engineering problems. It differs from nature-imitating (or biomimetic) and nature-integrated (or bio-incorporated) design, in that, instead of copying nature out of context, or incorporating living organisms, it focuses on using mechanistic features of natural systems, to create new technologies that may not superficially resemble their natural counterparts. In *On Growth and Form*, d'Arcy Thompson pioneered the vision that mathematics can serve as a language to describe biology [118]. Nature is full of mathematically definable patterns and features, such as logarithmic spirals, golden ratios, fractal geometry and Turing patterns, which are often related to specific physical–chemical mechanisms that, then, can provide guidelines for engineering. Living organisms have unique characteristics that lead to robust, adaptable properties with optimal resource management, which can be used as a source of inspiration for materials, product and process design. The biomedical field is only starting to benefit from nature-inspired approaches, with a vast array of research being conducted in tissue engineering and drug delivery [76], although most work still applies more narrowly biomimetic and bio-integrated approaches. Certain natural materials, such as mollusc nacre, gecko feet and shark skin have already had a major impact in creating novel tissue engineering technologies. The structures of natural membranes, cells and pathogenic microorganisms have provided inspiration to advance the field of drug delivery. However, once we embrace a systematic, mechanistic methodology, as advocated by the NICE approach, even more opportunities should arise to innovate in biomedicine, complemented and aided by parallel advances in additive manufacturing, computation and robotics.

## Data accessibility

This article has no additional data.
